# MRI quantification of intratumoral heterogeneity for predicting HER2-positive status in breast cancer: a retrospective multicenter study

**DOI:** 10.1186/s12885-026-15956-w

**Published:** 2026-04-22

**Authors:** Yong Zhou, Xinyi Wang, Fan Li, Hao Qin, Shiwen Chu, Ying Liu, Xiaojian Ma, Yan Dang, Wei Wei, Yun Zhang, Jie Ming

**Affiliations:** 1 Medical Imaging Center, Affiliated cancer Hospital of Xinjiang Medical University, Urumqi, Xinjiang 830011 China; 2 School of Electronics and Information, Xi’an Polytechnic University, Xi’an, 710048 China; 3 Department of Radiology, The Third Hospital of Mianyang, Sichuan Mental Health Center, Mianyang, 621000 China; 4 Special Needs Comprehensive Department, Affiliated cancer Hospital of Xinjiang Medical University, Urumqi, Xinjiang 830011 China; 5 Information Management and Data Center, Affiliated cancer Hospital of Xinjiang Medical University, Urumqi, Xinjiang 830011 China; 6 Scientific Research Office, Affiliated cancer Hospital of Xinjiang Medical University, Urumqi, Xinjiang 830011 China

**Keywords:** Magnetic resonance imaging, ITH, HER2-positive, Breast cancer

## Abstract

**Background:**

HER2 positive directly determines whether to use targeted drugs, but the existing methods are still lack of noninvasive methods. This study aims to predict HER2-positive status in breast cancer patients by quantifying Intratumoral heterogeneity (ITH) using pre-treatment MRI and to explore the association between ITH scores and HER2-positive response.

**Materials and methods:**

A multicenter retrospective study enrolled breast cancer patients who underwent pretreatment MRI at two hospitals, regardless of molecular subtype. ITH scores were derived from MRI radiomic features. Pearson correlation analysis was used to assess the relationship between ITH scores and HER2 status. The predictive value of ITH scores and age for HER2 positivity was also evaluated. Subgroup analyses by age and hospital were conducted to validate the association between HER2 positivity and related factors.

**Results:**

A total of 526 patients were included, with 294 from the first hospital and 232 from the second. The AUC for predicting HER2-positive status using ITH was 0.898, with a sensitivity of 0.827 and a specificity of 0.913. Additionally, an ITH + age combined model was constructed, which achieved an AUC of 0.898, sensitivity of 0.812, and specificity of 0.913 for HER2 positivity prediction in the validation set. Subgroup analysis revealed stable predictive efficacy across hospitals and improved discriminability for patients aged over 40.

**Conclusions:**

The ITH score is significantly associated with HER2-positive status in breast cancer and has potential as a non-invasive biomarker for predicting HER2-positive status. As an adjunct to pathological examination, it may support clinical decision-making but is not intended to replace tissue biopsy.

## Introduction

Breast cancer remains the leading cause of cancer incidence among women globally, making it one of the most common malignancies in females [1]. Human epidermal growth factor receptor 2 (HER2)-positive breast cancer is a highly aggressive subtype, and its treatment strategies have been a major focus in oncology research [2]. This subtype exhibits distinct biological behaviors, and although anti-HER2-targeted therapies [3] have improved patient prognosis, drug resistance remains a significant challenge. Therefore, accurately predicting the HER2-positive status in breast cancer patients prior to treatment is critical for developing personalized therapeutic strategies, and non-invasive prediction methods could offer significant advantages for clinical practice.

At present, the prediction of HER2-positive breast cancer has involved the fields of molecular biology, pathology and imaging. Although immunohistochemical tissue biopsy (IHC) remains the gold standard for determining HER2 status, it is invasive, prone to sampling bias, and cannot capture dynamic changes during treatment. Therefore, non-invasive imaging biomarkers such as ITH can serve as an auxiliary tool to support HER2 assessment, especially when biopsy is not feasible or repeated sampling is required. Although mammographic calcifications and ultrasound features of the breast have been established as traditional imaging predictors of HER2-positive status [4, 5], most of these features are qualitative. In contrast, quantitative imaging biomarkers, such as ITH, show unique advantages due to their reproducibility and non-invasiveness. As an important feature of tumor progression, high ITH is closely related to treatment resistance and poor prognosis in HER2-positive breast cancer [6]. However, traditional ITH assessment relies on multi-region tissue biopsy combined with genomic sequencing (or inferred from a single biopsy), or through histopathology [7], immunology [8], and other methods, which are not only invasive but also difficult to reflect the overall tumor heterogeneity and cannot achieve real-time dynamic monitoring during treatment. In comparison, MRI, with its multi-sequence and high soft tissue resolution characteristics, holds significant potential for the quantitative assessment of ITH [9–11].

Although MRI-based ITH quantification can reflect cell proliferation and microenvironment heterogeneity by analyzing tumor signal intensity, texture features, and so on, and should theoretically serve as an important method to explore the relationship between ITH and HER2 treatment response, current studies still have limitations: first, relatively few studies have directly used ITH scores as core features to predict HER2-positive subtypes [12]; second, some studies using traditional machine learning algorithms face problems of insufficient feature utilization and low model efficiency [13]; additionally, studies based on a single MRI sequence have not comprehensively explored the multidimensional information of ITH [14]. Therefore, it is necessary to develop a non-invasive imaging method capable of integrating multidimensional features, accurately quantifying ITH, and effectively predicting HER2 status.

Age is a critical biological variable in breast cancer, with 40 years being a widely recognized threshold distinguishing young patients who exhibit distinct genomic profiles [15]. Age-related differences in DNA repair pathways and mutation patterns may influence ITH and its interaction with molecular subtypes such as HER2-positive status [16]. Therefore, exploring the relationship between ITH and HER2 positivity across different age groups may provide valuable insights for personalized treatment strategies.

The purpose of this study is to take the ITH score as the core trait, use Pearson correlation coefficient test to examine the correlation between ITH score and HER2 status, and conduct a combined analysis of “ITH + age” to evaluate the association between the two and HER2-positive status, and use age as the basis for subgroup analysis to further explore the relationship between ITH and HER2-positive status under different age factors. In addition, this study also combines data from different hospitals for analysis to explore the association between ITH and HER2-positive status.

## Materials and methods

**Fig. 1:**
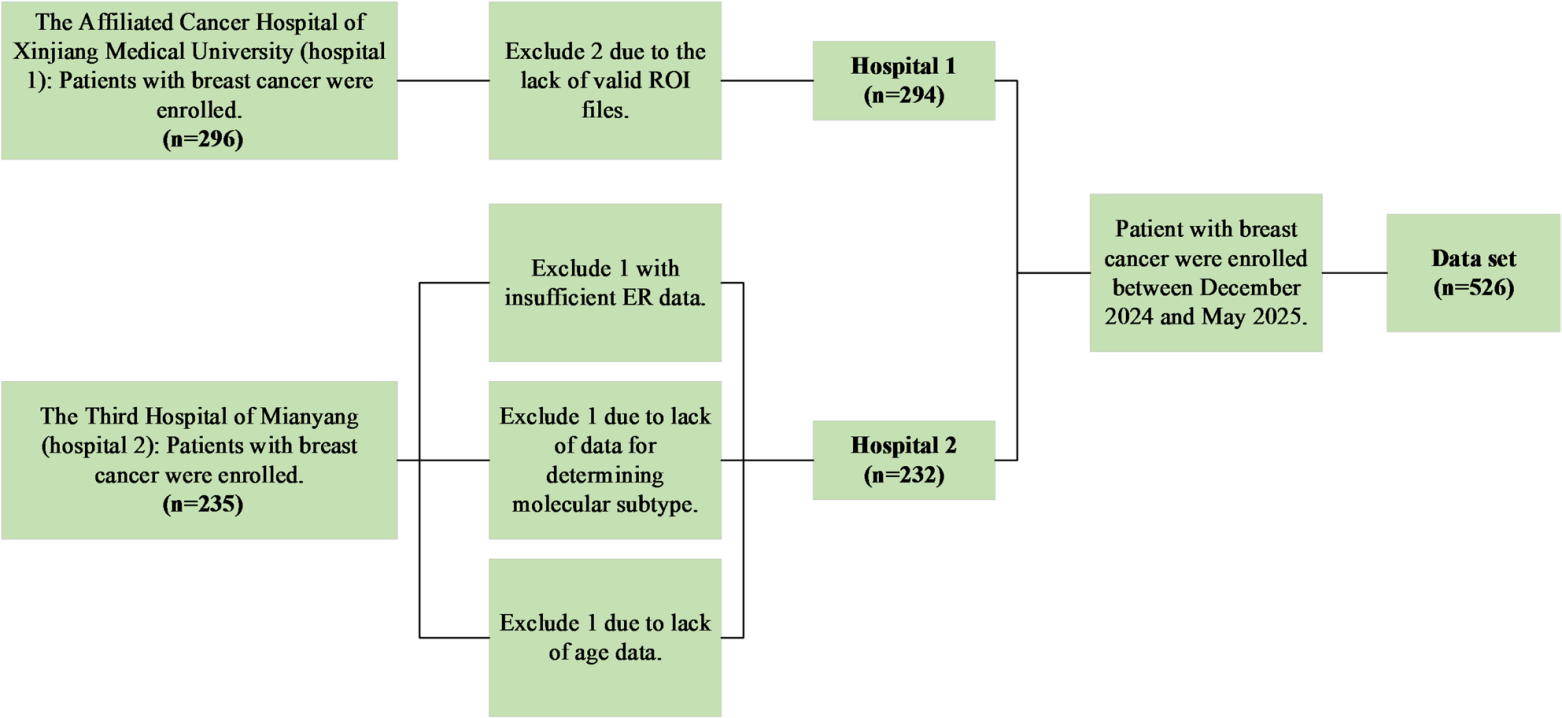
Patient inclusion flowchart shows the number of patients

This retrospective study included patient data from two hospitals (Fig. 1). Approval was obtained from the institutional review boards of both institutions, and the requirement for informed consent was waived.

### Eligibility criteria

Data were collected from breast cancer patients who underwent MRI at the Affiliated Cancer Hospital of Xinjiang Medical University (hospital 1) and The Third Hospital of Mianyang (Sichuan Mental Health Center, hospital 2), approval number: K-2,021,028. A total of 531 patients were initially screened. Among them, the first hospital had 296 cases and the second had 235 cases. Inclusion criteria were: (a) histopathologically confirmed breast cancer; (b) pre-treatment breast MRI performed at one of the two collaborating hospitals, including axial dynamic contrast-enhanced (DCE) sequences; (c) complete clinicopathological data. Exclusion criteria were: (a) absence of valid breast MRI data; (b) uncertain pathological results; (c) missing key clinical information; (d) stage IV (metastatic) breast cancer.

All included patients had non-metastatic, early-stage breast cancer (stages I-III). Patients who received preoperative neoadjuvant chemotherapy were permitted, provided that pre-treatment MRI was performed prior to therapy initiation. Mammography and ultrasound images were available in PACS for the majority of patients as part of routine clinical care; however, this study was designed specifically to investigate MRI-derived ITH, and mammography/ultrasound findings were not systematically analyzed. This study was designed specifically to investigate MRI-derived ITH; therefore, mammography and ultrasound findings were not systematically analyzed.

### Molecular subtypes and IHC staining criteria

ER and PR positivity were defined as ≥ 1% positively stained tumor nuclei by immunohistochemistry (IHC), following current clinical guidelines. HER2 positivity was defined as IHC 3+ (strong, complete membranous staining in > 10% of tumor cells) or IHC 2 + with confirmation of HER2 gene amplification by in situ hybridization (ISH), in accordance with established HER2 testing guidelines. Molecular subtypes were classified based on standard criteria: (i) Luminal A: ER + and/or PR+, HER2–, Ki-67 low(< 20%); (ii) Luminal B: ER + and/or PR+, HER2–, Ki-67 high(≥ 20%); or ER + and/or PR+, HER2+; (iii) HER2-enriched: ER–, PR–, HER2+; (iv) Triple-negative: ER–, PR–, HER2–. Ki-67 status was classified as low (< 20%) or high (≥ 20%) based on the commonly used threshold in clinical practice [17].

### MRI procedure and postprocessing

All MRI examinations were performed using a 3.0T scanner (SIGNA™ Premier, GE Healthcare). Dynamic contrast-enhanced MRI was acquired with a three-dimensional gradient echo sequence and fat suppression. Representative scan parameters were: TR 4 ms, TE 1.5 ms, flip angle 30°, slice thickness 3 mm, no interslice gap, field of view 28 × 28 cm, matrix 320 × 320, and NEX 1. The acquisition time per sequence was approximately 1.28 s. Tumor images for subsequent analysis are rapidly acquired, and these images correspond to the peak initial stage [first stage] of dynamic contrast-enhanced magnetic resonance imaging (DCE-MRI). All images were resampled to an isotropic resolution of 1 × 1 × 1 mm³ to ensure consistency across patients [18].

### Image segmentatio

Tumor segmentation was performed on the early phase (peak initial stage) images of dynamic contrast-enhanced magnetic resonance imaging (DCE-MRI). These images are acquired rapidly and best reflect the initial contrast uptake dynamics associated with tumor vascular heterogeneity.

The regions of interest for the tumors were manually outlined slice by slice by two physicians with over 5 years of experience in breast MRI diagnosis, using ITK-SNAP software. Both readers were blinded to the patients’ clinical and pathological information, including HER2 status. Outlines were drawn along the tumor margins, carefully avoiding areas of obvious necrosis, cystic change, or hemorrhage. Inter-observer consistency was evaluated by comparing the segmentations independently performed by the two radiologists.

For patients presenting with multifocal or central breast cancer (multiple lesions on the same side), the largest lesion (index lesion) is selected for delineation and subsequent ITH analysis. This approach is chosen because the index lesion typically represents the most aggressive tumor clone and is the primary target for clinical staging, biopsy, and treatment planning. This method is consistent with common practices in radiomics research.

### ITH score calculation

ITH refers to the genetic, phenotypic and functional diversity of cells in the same tumor is a key driver of treatment resistance and prognosis variation in breast cancer, especially in HER2 positive breast cancer, which may affect the sensitivity of targeted therapy.

To evaluate the heterogeneity of clustering patterns, we identified two quantifiable core indicators: one is the number of connected regions in cluster i (denoted as ${n_i}$), and the other is the area of the largest connected region within the cluster (denoted asX ${S_{i,\hbox{max} }}$). The variation patterns of these two indicators are directly related to the level of heterogeneity—for a single cluster, the more connected regions it contains and the smaller the area of the largest internal connected region, the higher the heterogeneity exhibited by the clustering pattern. In this study, ITHscore was used to quantify the overall degree of heterogeneity of the label map:

$${\text{ITH score=1-}}\frac{1}{{{S_{total}}}}\sum\limits_{{i=1}}^{V} {\frac{{{S_{i,\hbox{max} }}}}{{{n_i}}}} $$

**Fig. 2:**
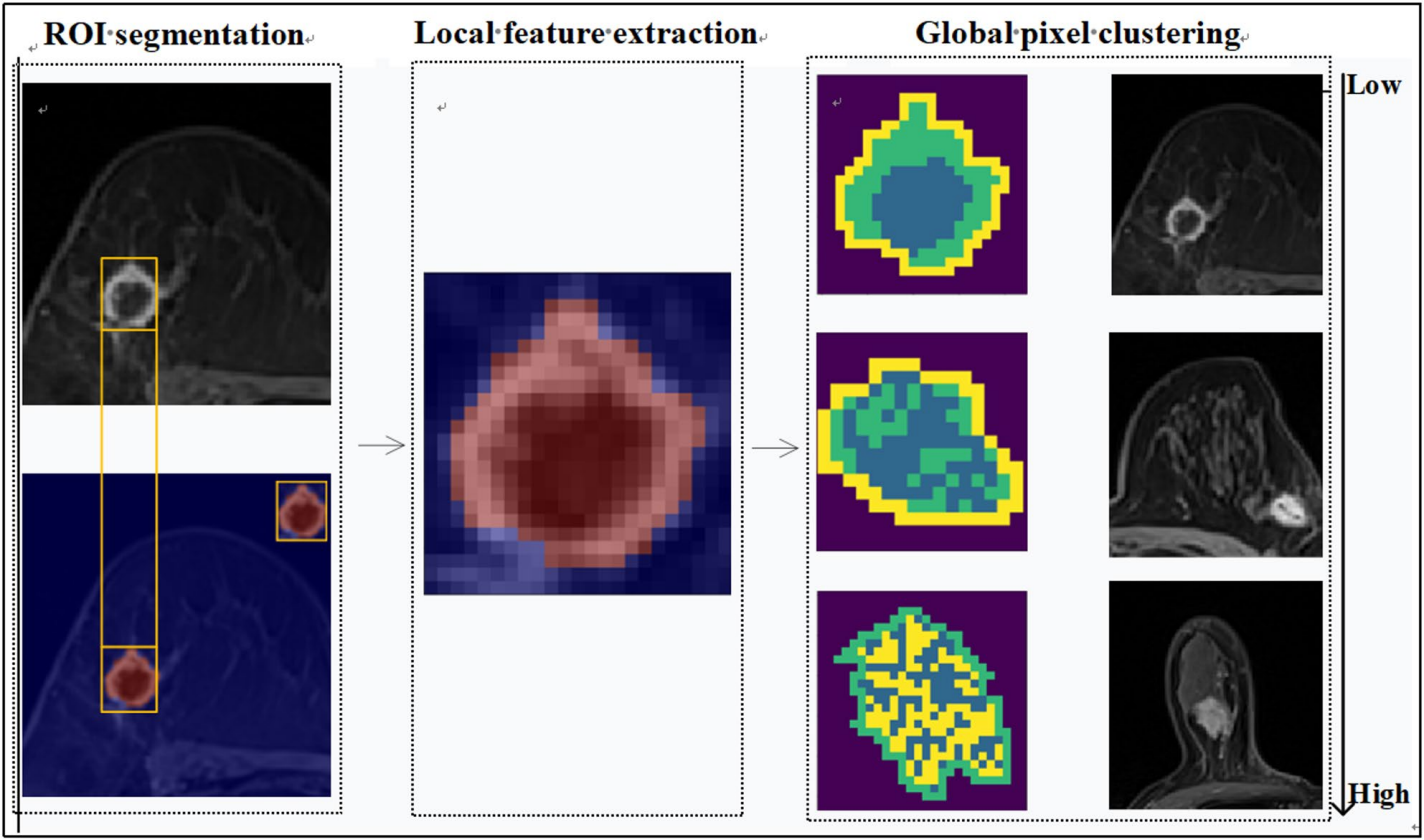
Schematic workflow of the study. Firstly, the tumor area depicted by hand is segmented, and the ROI is normalized. Secondly, use a 3 × 3 mm sliding window to extract local features (first-order and texture features). Thirdly, conduct unsupervised clustering based on local features, generate schematic diagrams to visualize the global distribution pattern, and calculate the intratumor heterogeneity (ITH) score. Fourth, taking the ITH score as the core feature, a HER2 positive prediction model based on the LightGBM algorithm was constructed. Finally, the performance of the model in predicting HER2-positive reactions and its potential biological significance were verified. Abbreviations: AUC, Area under the receiver operating characteristic curve; HER2, Human epidermal growth factor receptor 2; HR, Hormone receptor; ROI, Region of interest

In the calculation logic of ITHscore, V represents the total number of all clusters in the label map, and ${S_{total}}$ refers to the total area of the neoplasm. In terms of numerical range, the value range of ITHscore is from 0 to 1, and its numerical value is positively correlated with the degree of dispersion and heterogeneity of the label map: the higher the ITHscore, the more dispersed the spatial distribution of the label map, which in turn reflects the higher heterogeneity of cell composition and spatial arrangement in the region. Fig. 2 depicts the workflow of the study.

### Statistical analysis

This study used Python (version 3.9.5; Python Software Foundation) and R (version 4.3.1; R Foundation for Statistical Computing) for statistical analysis. Continuous variables were presented in different ways according to their distribution characteristics: those conforming to a normal distribution were presented as “mean ± standard deviation”, and those not conforming to a normal distribution were described as “median (interquartile range)”; categorical variables were expressed as “frequency (percentage)”.For the scenario of predicting HER2-positive status using ITH values, the area under the receiver operating characteristic curve (AUC), sensitivity, and specificity were employed as core evaluation metrics. Specifically, AUC quantifies the ability of ITH values to distinguish between HER2-positive and HER2-negative samples, sensitivity reflects the capacity to identify HER2-positive samples, and specificity represents the ability of ITH to exclude HER2-negative samples. Statistical significance was determined with *P* < 0.05 as the criterion, that is, when the *P* value was less than 0.05, the difference was considered statistically significant.

First, the Pearson correlation coefficient was used to analyze the linear relationship between ITH and HER2. The core function is to calculate the R value and *P* value: among them, the R value is used to determine the strength and direction of the linear association between the two variables. The *P*-value is used to determine whether the linear association reflected by the R-value is statistically significant through probability (usually with *P* < 0.05 as the threshold, the smaller the value, the less likely the association is to be caused by random error). The combination of them is applicable to the quantification of the linear association between ITH and HER2, direction determination and significance verification, providing a basis for subsequent association prediction research.

Second, two analyses were conducted to compare the predictive performance across different variable dimensions: (1) A univariate analysis of the entire sample, which solely evaluated how well individual ITH values predict HER2 positivity, calculating key metrics including AUC, sensitivity (true positive rate), and specificity (1 - false positive rate); (2) A concurrent multivariate analysis, which assessed the combined predictive power of “ITH value + age” for HER2 positivity, with the same key metrics computed. Furthermore, by plotting ROC curves for both analytical approaches [19], the discriminative ability of individual ITH values versus the “ITH value + age” combination in identifying HER2-positive samples was visually demonstrated, enabling clear comparison of performance differences between univariate and multivariate prediction models.

Finally, subgroup analyses were conducted based on the patients’ ages (Age ≤ 40 and Age > 40) to calculate the AUC, sensitivity and specificity of the ITH model in each group, in order to evaluate the differences in the impact of ITH scores on the HER2 positive status in different subgroups. This study also analyzed data from different hospitals, calculated the above indicators in the same way, and plotted the ROC curve to explore the association between ITH and HER2 positive status. To assess the generalizability of the model, data from Hospital 1 (*n* = 294) were used as the training set, while data from Hospital 2 (*n* = 232) were used as an independent external validation set to evaluate the model’s final performance.

## Result

Data from 531 women with breast cancer were initially retrieved from two hospitals. Among these, 526 patients with early-stage (I–III) breast cancer were included in the final analysis (median age, 48 years [interquartile range, 42–55 years]). Among these, 21 (4%) had stage I, 263 (50%) had stage II, and 242 (46%) had stage III disease. Preoperative neoadjuvant chemotherapy was administered to 268 patients (51%), all of whom underwent pre-treatment MRI before starting therapy. Of these, 335 patients (64%) were estrogen receptor (ER)-positive and 191 (36%) were ER-negative; 350 (67%) were progesterone receptor (PR)-positive and 176 (33%) were PR-negative (Table 1). The median ITH score for the overall cohort was 0.6052 (range, 0-0.9998). Stratified by molecular subtypes, the median ITH scores were as follows: HR+/HER2-: 0.4606 (range, 0-9813); HR+/HER2+: 0.8317 (range, 0.0259–0.9998); HR-/HER2+: 0.8643 (range, 0.209–0.9989); HR-/HER2-: 0.1272 (range, 0-9072).

**Table 1:** Clinical and pathological characteristics information of the patient

Characteristic	Data set *n* = 526
Age (y)^†^	48 (42–55)
ER status	
Negative	191 (36)
Positive	335 (64)
PR status	
Negative	176 (33)
Positive	350 (67)
HER2 status	
Negative	335 (64)
Positive	191 (36)
Ki-67 status	
Low (< 20%)	73 (14)
High (≥ 20%)	453 (86)
Unknown	0 (0)
Molecular subtype	
HR + /HER2 +	118 (22)
HR + /HER2 -	266 (51)
HR - /HER2 +	73 (14)
HR - /HER2 -	69 (13)
Menopausal status	
Premenopausal	268(51)
Postmenopausal	258(49)
Histological grade	
Grade 1	26(5)
Grade 2	352(67)
Grade 3	148(28)
Clinical disease stage	
Stage Ⅰ	21(4)
Stage Ⅱ	263(50)
Stage Ⅲ	242(46)

This study focused on MRI-derived ITH, mammographic and ultrasound features were not systematically analyzed

*Abbreviations*: *HER2* human epidermal growth factor receptor 2, *ER* Estrogen Receptor, *PR* Progesterone Receptor, *Ki-67* Ki-67 Antigen, HER2−, HER2 negative, HER2+, HER2 negative

^†^Data are medians, with IQRs in parentheses

### Overall analysis

**Fig. 3:**
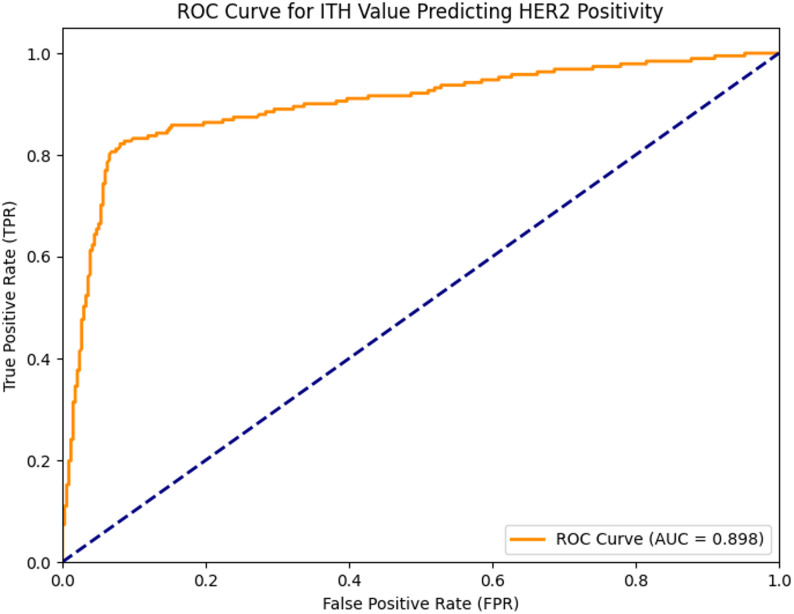
Receiver operating characteristic curve for ITH value predicting HER2 positivity

**Fig. 4:**
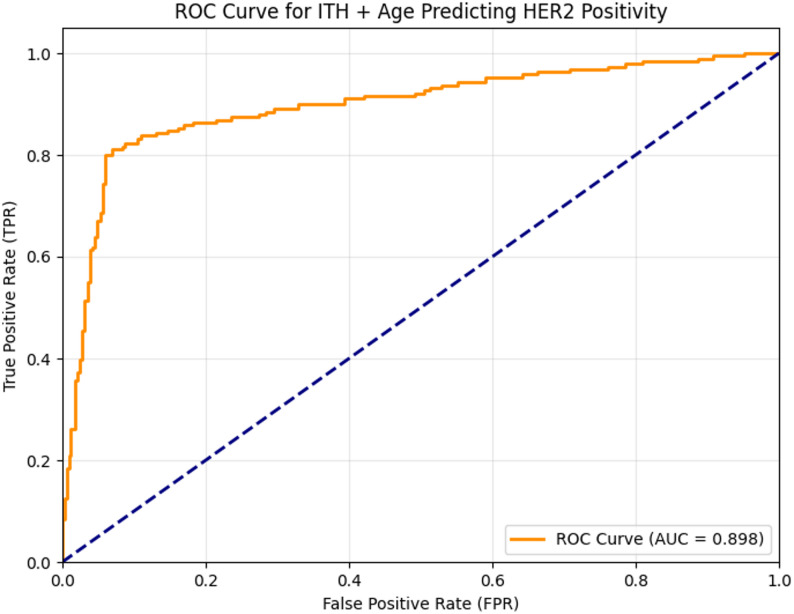
Receiver operating characteristic curve for ITH + Age predicting HER2 positivity

**Fig. 5:**
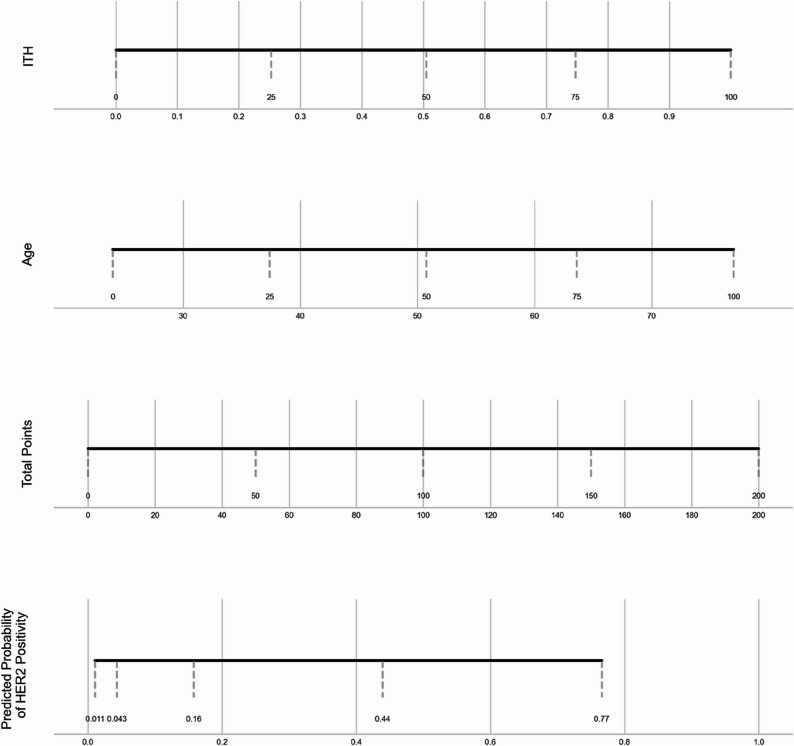
Nomogram for HER2 prediction based on ITH and Age

In the overall data of 526 patients included in the analysis, the correlation between ITH and HER2 was first analyzed. The results showed that the correlation coefficient between ITH and HER2 was 0.6340, indicating a moderately strong positive linear association between ITH and HER2, with *p* < 0.05, and the positive linear association between ITH and HER2 was of extremely strong statistical significance. When using the (ITH score to predict HER2 positive, the area under the curve (AUC) reached 0.898 (Fig. 3), with a sensitivity of 0.827 and a corresponding specificity of 0.913. To better predict the HER2 positive status, we further combined ITH value with age for analysis. In the overall data evaluation of 526 patients, the area under the curve (AUC) of this combined prediction method was 0.898 (Fig. 4), with a sensitivity of 0.812 and a specificity of 0.931. The HER2-positive prediction model integrating ITH and age data, built using the training set, achieved an AUC of 0.808 in the independent external validation set. The corresponding nomogram is shown in Fig. 5.

### Analysis by hospital

**Fig. 6:**
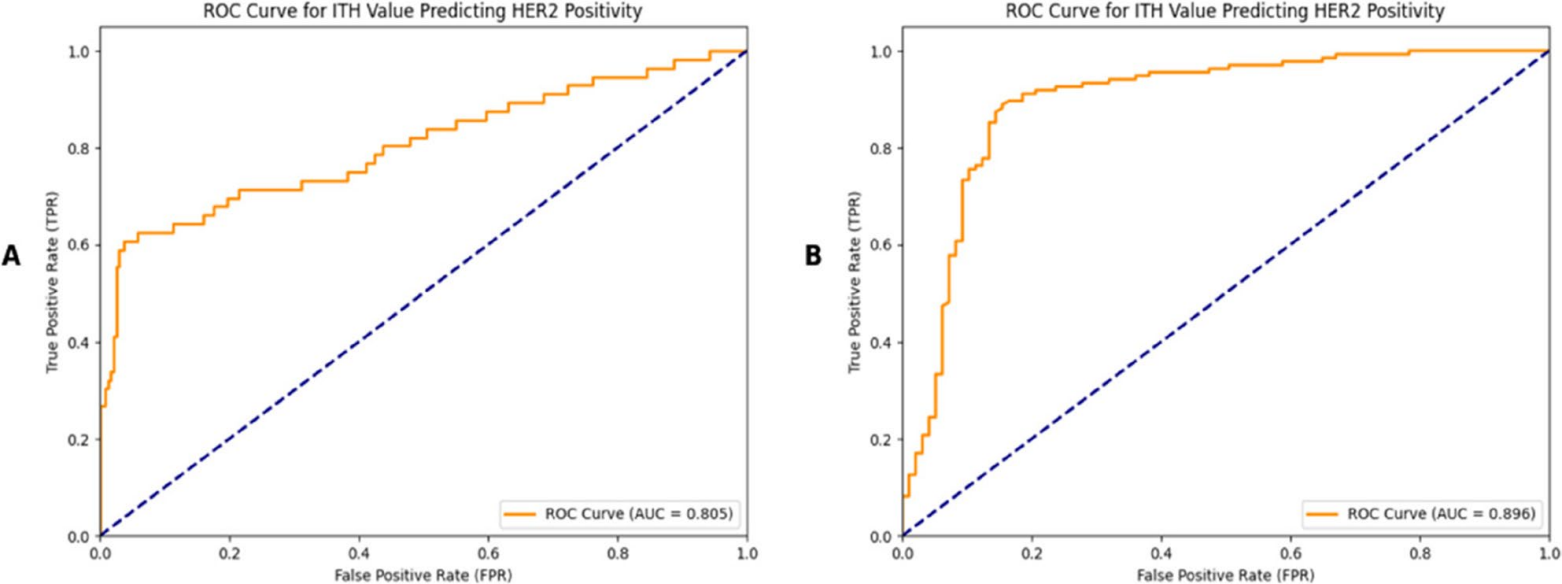
Receiver operating characteristic curve for ITH value predicting HER2 positivity in different hospitals: **A** Receiver operating characteristic curve for prediction of HER2 positivity based on ITH in patients from hospital 1. **B** Receiver operating characteristic curve for prediction of HER2 positivity based on ITH in patients from hospital 2

In the first hospital (*n* = 294; 56 HER2-positive, 238 HER2-negative), the ITH score yielded an AUC of 0.805, with a sensitivity of 0.607 and a specificity of 0.962. In the second hospital (*n* = 232; 135 HER2-positive, 97 HER2-negative), the AUC was 0.896, with higher sensitivity (0.889) and specificity (0.845). These findings demonstrate consistent predictive performance across independent institutional datasets (Fig. 6).

### Subgroup analysis by age

**Fig. 7:**
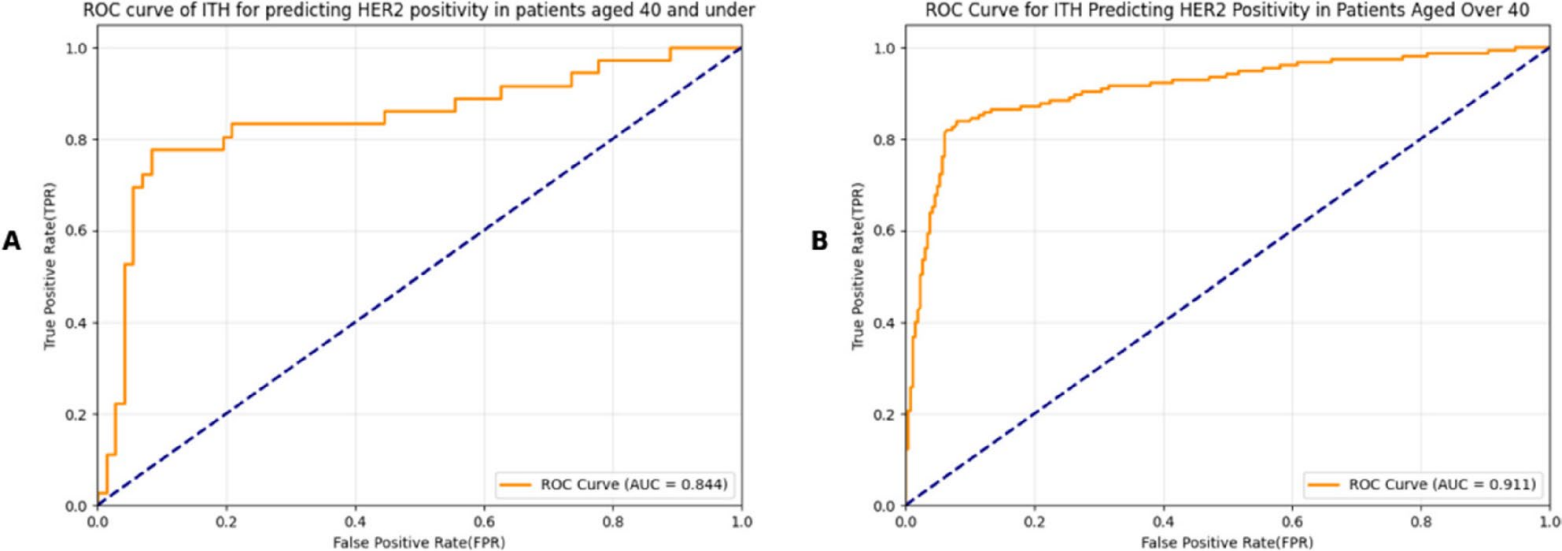
Receiver operating characteristic curve for ITH value predicting HER2 positivity in different age groups: **A** Receiver operating characteristic curve for prediction of HER2 positivity based on ITH in patients from aged ≤ 40 years. **B** Receiver operating characteristic curve for prediction of HER2 positivity based on ITH in patients from aged > 40 years

When stratified by age, patients ≤ 40 years (*n* = 108) showed an AUC of 0.844, with a sensitivity of 0.778 and a specificity of 0.917. Patients > 40 years (*n* = 418) demonstrated an AUC of 0.911, with a sensitivity of 0.839 and a specificity of 0.920. The older age group thus exhibited slightly better discriminative performance (Fig. 7).

## Discussion

This study quantitatively assessed ITH using pre-treatment MRI and determined that the ITH score could serve as a potential imaging biomarker for HER2 prediction, demonstrating excellent discriminative ability (AUC: 0.898). Lower ITH scores were associated with more favorable treatment responses and milder tumor biological behavior, which aligns with the theory that tumor heterogeneity drives therapy resistance. By integrating age as a clinical factor, the model’s diagnostic specificity was further improved. The findings of this study provide a new direction for addressing the current clinical challenge of the lack of efficient, non-invasive methods for assessing HER2-positive status [20].

ITH arises from the diverse in cell composition and spatial distribution within the tumor microenvironment [21, 22]. Imaging can non-invasively characterize these phenotypic variations at multiple spatial scales [23]. In HER2-positive breast cancer, heterogeneous HER2 protein expression and subclonal evolution lead to different intratumoral subregions [24], while the multiparametric nature of MRI provides an effective platform for ITH quantification [25]. Based on this, this study employed an unsupervised clustering method to achieve ITH quantification by integrating local radiomic features with global pixel-level patterns, without relying on large-scale datasets. Previous studies have insufficiently explored the value of ITH scoring, and the adaptability of algorithms in different clinical settings remains a challenge [26]. This study found that the proportion of high ITH scores was significantly higher in HER2-positive patients. Consistency between the two centers in terms of MRI systems, protocols, and acquisition parameters maximized potential technical differences, and the distribution of ITH was comparable. This indicates that ITH reflects not only morphological diversity but also HER2-driven biological behavior, providing a theoretical basis for its clinical application.

Various radiomics models have been developed in the field of breast cancer imaging to predict HER2 expression. For example, Zhang et al. [27] used multiparametric MRI to distinguish HER2 expression subgroups, with an AUC of 0.779–0.889; Cao et al. [28] integrated cross-modal radiomics features, achieving an AUC of 0.822. Studies by Lafcı, Qin, and Xie et al. [29–31] validated the value of imaging heterogeneity, but their AUCs were only 0.702–0.820, and most focused on single-dimensional feature extraction, failing to achieve a global quantitative analysis of heterogeneity. Unlike these studies, this research focuses on the intrinsic association between ITH and HER2 status, using a dedicated quantitative feature extraction strategy that combines local radiomics features with global pixel-level clustering patterns, thereby more accurately reflecting tumor spatial distribution and biological heterogeneity characteristics closely associated with HER2 amplification/overexpression.

Other technical methods are also widely used for predicting HER2 status. Deep learning models have achieved AUC values ranging from 0.78 to 0.85 through techniques such as multi-sequence MRI fusion and visual Transformer models [32, 33]. However, these models have significant “black box” issues, are highly dependent on large-scale annotated data and standardized acquisition protocols, and have poor generalizability across multiple centers, making clinical translation difficult. Mammography and ultrasound, as first-line screening methods, infer HER2 status through qualitative assessment of morphological features [34, 35]. Although they are simple to operate and low-cost, diagnostic results are greatly influenced by the subjective experience of radiologists and cannot capture the intrinsic biological heterogeneity of tumors at the molecular and cellular levels; the AUC values for HER2 prediction are usually below 0.80 [35, 36]. We acknowledge that these imaging semantic features provide important clinical information, but the goal of this study is different: to evaluate whether MRI-derived quantitative intratumor heterogeneity (ITH) can serve as a non-invasive biomarker for HER2 status. Unlike qualitative features, ITH provides a continuous, reproducible measurement of tumor heterogeneity. The excellent performance of ITH (AUC 0.898) indicates that quantitative heterogeneity assessment captures biological information associated with HER2 status. These two methods are complementary rather than competitive.

In comparison, the MRI-based ITH quantification method proposed in this study demonstrates a clear advantage in predictive performance. The core advantages are reflected in three aspects: first, unlike previous radiomics studies that analyze single-dimensional features [29–31], the dedicated quantitative feature extraction strategy in this study more comprehensively reflects the spatial distribution and biological heterogeneity of tumors; second, by avoiding deep learning’s over-reliance on large-scale datasets and the subjective qualitative assessment of mammography and ultrasound, it achieves objective and reproducible ITH quantification, with the model performing consistently across two independent hospital cohorts and showing good multi-center adaptability; third, incorporating age as a clinical auxiliary factor optimizes discrimination ability, making the predictive results more consistent with clinical-pathological patterns, especially for patients over 40 years old with more pronounced tumor biological characteristics. In addition, ITH quantification based on peak-phase images of high soft tissue resolution DCE-MRI can accurately capture microcirculation and cell proliferation heterogeneity associated with HER2 status. Compared with single-sequence MRI studies, it can establish a more direct and close imaging-molecular subtype correlation.

In addition to predicting HER2-positive status, the ITH score also shows potential as a biomarker for predicting the efficacy of neoadjuvant chemotherapy. Huang et al. [17] developed a nomogram model that combines the ITH score with clinicopathological variables. In a cohort of 1,448 patients from nine centers, the model predicted pCR with an AUC of 0.79–0.82 and demonstrated that lower nomogram scores were associated with poorer recurrence-free survival. These findings collectively support that ITH quantification can serve as a dual-function biomarker with both molecular subtype and efficacy prediction value, providing direct guidance for individualized treatment decisions: patients with high ITH scores may consider intensified treatment regimens to overcome chemoresistance, while patients with low ITH scores may consider de-escalated treatment.

This study has certain limitations. First, due to the retrospective multicenter design of the study, the sample size was relatively limited, and some clinicopathological variables (such as histological type and nuclear grade) were not consistently available and therefore could not be included in the analysis, which may introduce selection bias. Future research should conduct prospective studies using standardized data collection methods for validation. Second, the assessment of ITH relies on manual tumor delineation, which may not fully capture three-dimensional and multiparametric heterogeneity. Future studies should consider automated whole-tumor segmentation and deep learning feature optimization. Third, this study did not include an analysis of breast calcifications or ultrasound features because the study design focused on MRI-based ITH, and other imaging data were not systematically collected. Finally, ITH quantification was only based on peak-phase DCE-MRI images and did not include DWI-derived features, limiting the comprehensiveness of heterogeneity assessment. Future studies should integrate DWI and other multiparametric MRI to more comprehensively characterize ITH.

## Conclusion

In conclusion, this study demonstrates that MRI-derived ITH scores are strongly associated with HER2-positive status in breast cancer. While ITH has not yet been integrated into predictive models, our findings provide a theoretical and practical foundation for incorporating ITH into future modeling efforts. Such integration is expected to improve predictive accuracy and support precision diagnosis and personalized treatment of breast cancer.

While the ITH score demonstrates strong association with HER2-positive status and shows promise as a non-invasive imaging biomarker, it is not intended to replace tissue biopsy. Rather, it may serve as a complementary tool to support clinical decision-making, particularly in scenarios where biopsy is challenging or repeated assessment is needed.

## Data Availability

The datasets generated during the current study are available from the corresponding author on reasonable request.

## Abbreviations

AUC: Area under the receiver operating characteristic curve
HER2: Human epidermal growth factor receptor 2
HR: Hormone receptor
ITH: Intratumoral heterogeneity
ROC: Receiver operating characteristic curve
